# The Metameric Echinoderm

**DOI:** 10.1093/iob/obae005

**Published:** 2024-03-01

**Authors:** R L Turner

**Affiliations:** Department of Ocean Engineering and Marine Sciences, Florida Institute of Technology, Melbourne, FL 32901-6975, USA

## Abstract

Animal phyla are distinguished by their body plans, the ways in which their bodies are organized. A distinction is made, for example, among phyla with bodies of many segments (metameric; e.g., annelids, arthropods, and chordates), others with completely unsegmented bodies (americ; e.g., flatworms and mollusks), and a few phyla with bodies of 2 or 3 regions (oligomeric; e.g., echinoderms and hemichordates). The conventional view of echinoderms as oligomeric coelomates adequately considers early development, but it fails to recognize the metameric body plan that develops in the juvenile rudiment and progresses during indeterminate adult growth. As in the 3 phyla traditionally viewed to be metameric (annelids, arthropods, and chordates), metamery, or metamerism, in echinoderms occurs by (1) subterminal budding of (2) serially repeated components of (3) mesodermal origin. A major difference in most echinoderms is that metamery is expressed along multiple body axes, usually 5. The view of a metameric echinoderm might invite new discussions of metazoan body plans and new approaches to the study of morphogenesis, particularly in comparative treatments with annelids, arthropods, and chordates.

## German

Tierstämme zeichnen sich durch ihren Körperbauplan, das heist, die Art und Weise aus, wie ihre Körper organisiert sind. Man unterscheidet beispielsweise Stämme mit Körpern aus vielen Segmenten (metamerer; z. B. Ringelwürmer, Arthropoden, Akkordaten), andere mit völlig unsegmentierten Körpern (amerer; z. B. Plattwürmer, Mollusken) und einige wenige Phyla mit Körpern aus zwei oder drei Regionen (oligomerer; z. B. Stachelhäuter, Hemichordaten). Die herkömmliche Sichtweise von Stachelhäutern als oligomere Coelomate berücksichtigt die frühe Entwicklung angemessen, berücksichtigt jedoch nicht den metameren Körperplan, der sich im jugendlichen Rudiment entwickelt und während des unbestimmten Erwachsenenwachstums fortschreitet. Wie bei den drei Phyla, die traditionell als metamer angesehen werden (Anneliden, Arthropoden, Chordaten), erfolgt Metamerie bei Stachelhäutern durch (1) subterminale Knospung von (2) seriell wiederholten Komponenten (3) mesodermalen Ursprungs. Ein wesentlicher Unterschied bei den meisten Stachelhäutern besteht darin, dass die Metamerie entlang mehrerer Körperachsen, normalerweise fünf, ausgedrückt wird. Die Sicht auf einen metameren Stachelhäuter könnte zu neuen Diskussionen über Metazoen-Körperpläne und neue Ansätze zur Untersuchung der Morphogenese führen, insbesondere bei vergleichenden Behandlungen mit Ringelwürmern, Arthropoden und Chordaten.

## Introduction

The Animal Kingdom (Metazoa) is a menagerie of about 3 dozen phyla, each phylum defined by a unique body plan. Over the 41-year history of 7 editions of Barnes's textbook *Invertebrate Zoology*, the number of recognized phyla increased from 25 to 33, more by elevation of lower groups to phylum status than to the discovery of new groups (Barnes 1963; Ruppert et al. 2004). More recent textbooks give a range of 31–39 phyla (Valentine 2006; Nielsen 2012; Pechenik 2015; Brusca et al. 2023). Some phyla (e.g., arthropods, mollusks, chordates, and annelids) include thousands of species of animals with large bodies, and they impact our lives often and directly; but many phyla, unfamiliar to the layperson, are low in species diversity, are microscopic, or live in places rarely visited by humans. Yet, all are distinguished by their body plans, a concept that originated in the early 1800s (Holland et al. 2015). The definition and evolution of metazoan body plans remain active areas of study and debate in comparative biology and phylo-evo-devo today (Raff 1996; Arthur 1997; Minelli 2003a, 2009; Willmore 2012; Fusco 2019; Isaeva and Rozhnov 2021; Nanglu et al. 2023).

Naturalists since the mid-1800s have sought to organize metazoan diversity into superphyletic taxa that reflect the authors’ perceptions of phylogenetic relationships (Clark 1964; Willmer 1990). These efforts gave rise to many names familiar to generations of students of zoology: Acoelomata, Articulata, Aschelminthes, Deuterostomia, Gephyrea, Radiata, Spiralia, Triploblastica, Trochozoa, Zoophytes, and others. The criteria for superphyletic ranking have included patterns of symmetry, cleavage, and germ-layer formation; kinds of larval stages, body cavities, and skeletons; divisions of the body; and recent evidence from molecular biology, genetics, and ultrastructure. The schemes for organizing animal phyla might now rival the Metazoa in diversity and are themselves the subject of categorizing by some authors (Willmer 1990).

One criterion used in many phylogenetic schemes is the division of the body into “segments.” The premiere example is Hadži's (1963) clustering of Metazoa into Ameria, Polymeria, Oligomeria, and Chordonia. His Ameria included the unsegmented “flatworms,” ribbon worms, cnidarians, “aschelminths,” and mollusks. Annelids, echiurans, arthropods, and onychophorans—with many segments or presumed vestiges of them—comprised the Polymeria. Members of the Oligomeria and Chordonia had bodies with 2 or 3 parts derived from subdivision of the coelom early in development. Hadži's Oligomeria consisted of echinoderms, the “lophophorates,” peanut worms, pogonophorans, arrow worms, and hemichordates; the Chordonia were the chordates. Among the Polymeria, the annelids and arthropods are 2 groups generally recognized to demonstrate metamery, a special case of the serial repetition of segments; this condition might prevail also in some minor phyla (Telford et al. 2019). The vertebrates (by some authors, more broadly, the chordates) also are regarded to be metameric in the segmentation of the anteroposterior axis despite their frequent inclusion with oligomeric phyla such as echinoderms.

The most thorough analysis of metamery is Clark's (1964)  *Dynamics in Metazoan Evolution*. Clark described the morphological expression of metamery among animals, its functional significance in locomotion, and its phylogenetic implications. His book has been followed by several discussions of metamery in textbooks on comparative invertebrate biology. Except for 2 authors (Haeckel 1866,^1889^; Gislén 1930) whose works long predate Clark's book, advanced and elementary textbooks on animal and general biology state either that echinoderms are unsegmented (or are oligomeric) or that annelids, arthropods, and chordates are the only major groups with metamery. The situation is similar in primary and review literature. For example, Davis and Patel (1999) wrote that segments of the latter 3 phyla are “traditionally. . . accorded special significance as examples of metamerism.” Seaver (2003) also treated these 3 as the “overtly segmented” or “eusegmented” metazoan phyla, and Chipman (2019) treated them as the 3 “fully segmented” phyla. The exclusion of echinoderms from treatments of metamery has puzzled me for 50 years of research and teaching. The objective of the present work is to establish the metameric organization of this “non-traditional” fourth major group, the echinoderms.

## Essentials of metamery

**Fig. 1:**
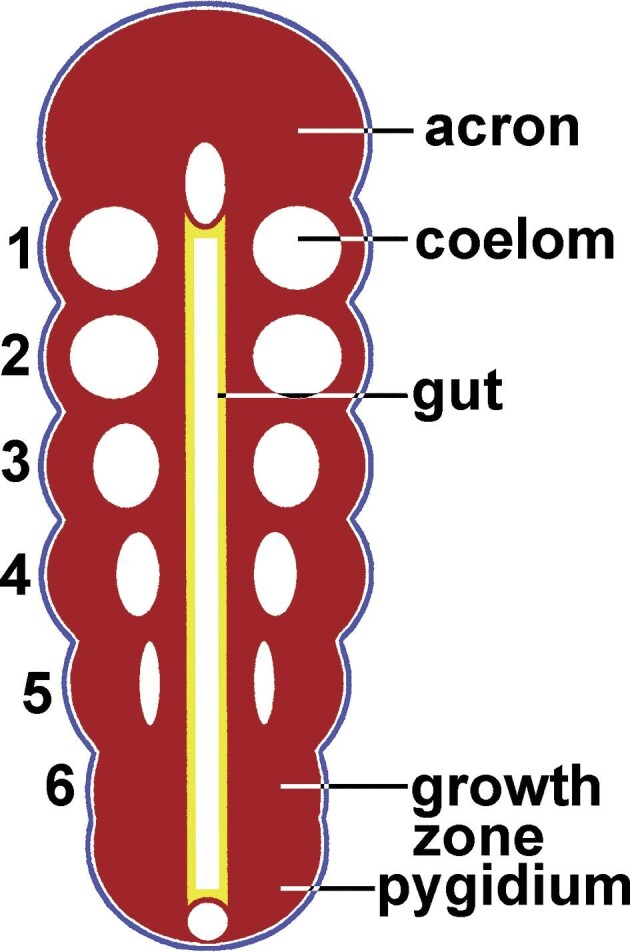
Schematic of a representative metameric metazoon. The anterior (acron) and posterior (pygidium) regions of the animal become increasingly separated by the insertion of segments (nos. 1–6) between them in a growth zone immediately anterior to the pygidium. The oldest segment (no. 1) lies behind the acron, the youngest (no. 6) anterior to the pygidium. This pattern of growth is called subterminal, penultimate, or teloblastic budding. Segments have parts of the coelom within tissue of mesodermal origin. The coelomic lining is not colored because metazoan coeloms can derive from embryonic ectoderm (blue), endoderm (yellow), or mesoderm (red).

A metameric body plan (Fig. 1) is defined by 3 conditions (Willmer 1990): serial repetition of body parts, involvement of the mesoderm, and subterminal addition of new segments. Clark (1964) recognized the first 2 conditions but still regarded “metamerism” to be a rather vague term. Russel-Hunter (1969) and others (e.g., Chaudonneret 1979; Balavoine and Adoutte 2003) later recognized the third condition, arising early in development and defining the term more restrictively. Satisfaction of these 3 conditions does not imply that the occurrence of metamery in different phyla is homologous (Moore and Willmer 1997; Davis and Patel 1999; Scholtz 2002; Minelli 2003a), but Balavoine and Adoutte (2003) argued for homology from a metameric urbilaterian.

Many animal groups have bodies with repeated parts along the longitudinal axis. Serial repetition might involve gonads, muscles, nerves, excretory organs, gills, limbs, cuticle, endoskeleton, gastric pouches, body cavities (including septation), and blood vessels. Serial repetition is routinely demonstrated in the dissection of earthworms, crayfish, and dogfish by students of comparative anatomy, and it is observable without dissection in the arrangement of associated appendages or the annulation of the body in arthropods, earthworms, and tapeworms. Such repetition of parts is the essential element of all definitions of metamery. On this basis, Hyman (1955) rejected use of the term “three-segmented” and “oligomerous” in reference to deuterostome and other oligomeric phyla because the 3 regions of the body do not include repeated elements of organ systems that correspond with the 3 sets of coelomic pouches. Clark (1964) agreed with Hyman's assessment but not so vehemently. (Oligomery is defined and discussed further below.)

The mesodermal basis for metamery and its association with the coelom were emphasized by Clark (1964). His clarification of the term eliminated, for example, flatworms and ribbon worms with serially repeated gastric pouches (endodermal features) and nautiloids and rotifers with segmented shells or cuticles (epidermal secretions) as examples of metamery. Repetition in the musculature is the primary defining feature because of the involvement of metamery in locomotion by action of muscles on a hydrostatic skeleton (annelids), a cuticle (arthropods), or an endoskeleton (vertebrates). Septation of the coelom is not, however, a requirement for metamery (Clark 1964; Willmer 1990). Other body parts of ectodermal or endodermal origin might be serially repeated in conjunction with segmentation of the musculature.

Subterminal (penultimate) budding is the third element of metamery (Fig. 1). A diagram of a metatrochophore or a metanauplius often is used to illustrate the budding zone (teloblast) just anterior to the perianal region, or pygidium. As a result of this pattern, the youngest segments are posterior, though subterminal; and the oldest are anterior, near the head, or acron. Willmer (1990) alluded to subterminal budding as she concluded her definition of metamery: “with an organized developmental origin for this repetition.” In contrast, the reproductive budding, or strobilation, of tapeworms, flatworms, and cnidarian polyps is basal.

## The oligomeric echinoderm

**Fig. 2:**
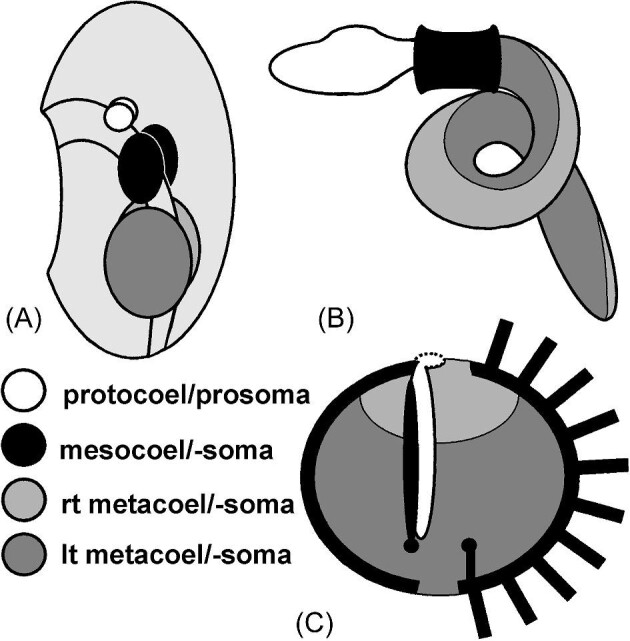
Organization of the oligomeric body plan. (**A**) In the embryo or larva, the coelom divides into 3 paired or unpaired cavities: protocoel, mesocoel, and metacoel. (**B**) In an acorn worm, the 3 body regions that contain the three coelomic cavities are easily recognized as the proboscis (prosoma with contained protocoel), collar (mesosoma with mesocoel), and trunk (metasoma with metacoel). (**C**) Oligomeric organization in a sea urchin, as a representative echinoderm, is obscured by expansion of the mesosoma around the other 2 regions. The prosoma includes the perforated madreporite at the surface and the axial organ descending from it. The perivisceral coelom, mostly comprised of the left metacoel, occupies much of the internal space along with viscera (not shown). The mesosoma has an internal component (stone canal) associated with the axial organ. The stone canal connects by a ring canal to 5 (one shown here) radial canals, which supply the externally visible parts of the water-vascular system, seen as podia extending from the body surface. Five paired rows (ambulacra) of podia alternate with 5 interambulacra; these 10 sections of the body form the body wall (external mesosoma). Much of the body wall is calcified as ossicles, pieces of the articulated skeleton of mesodermal origin.

The prevailing view of the echinoderm body plan has long been that of an oligomeric (archimeric, trimeric, 3-segmented) coelomate, a feature shared with other deuterostomes and with lophophorates (Clark 1964; Nielsen 2012). The oligomeric condition arises early in development with the division of the coelom into 3 linearly arranged compartments and their associated body regions (Fig. 2A–B): an anterior protocoel (paired or unpaired) of the prosoma; paired (right and left) mesocoels of the mesosoma; and paired posterior metacoels of the metasoma. This archetypic arrangement is highly modified among the several oligomeric phyla, and the origin, sequence of appearance, and fates of the compartments during embryogenesis and later development vary widely within the Echinodermata (Hyman 1955; Giese et al. 1991). Growth of the juvenile rudiment of the left side of the larval body in many echinoderms is correlated with the regression or disappearance of the protocoel and mesocoel on the right side of the larva and expansion of compartments on the left. In particular, the left mesocoel (hydrocoel) and mesosoma produce the water-vascular system (Fig. 2C), one of the most definitive characteristics of the phylum. This pattern can be highly altered in echinoderms without a feeding larva (e.g., Morris 2007). The outcome of embryo- and larvigenesis is the stacking of the coeloms along an anteroposterior (Mooi and David 2008) or oral-aboral axis (Formery et al. 2023).

## The case for metamery of the echinoderm body

Metamery in echinoderms is within the experience of novice sorters of marine benthos. Polychaete systematists and other museum specialists find vials of brittlestar arms labeled “polychaete” (or “polychaete fossils”; Simmons, pers. comm.) in processed benthic samples and even accessioned into museums (Holthe 1992; Cochrane, Fauchald, Fournier, Harris, ten Hove, Mah, Martin, Petersen, and Platell, pers. comm.). Some students of invertebrate zoology have been tricked by clever teachers who present them with brittlestar arms separated from their disks and who allow their students to stumble into the conclusion that the sample is a mass of polychaetes (Lares and Robertshaw, pers. comm.). Some supervisors in benthic ecology purposely train their apprentices to avoid this mistake (Culter and Parker, pers. comm.). A commonly suggested detection method includes a distinction between crunchy (brittlestar arms) and soft (polychaete) bodies (Cochrane, Fauchald, Harris, Leverone, and Parker, pers. comm.). The errors are due to the serial repetition of parts (called “segments” by brittlestar specialists; Hendler 2018) along the brittlestar arm and the superficial resemblance of arm spines and podia/tube feet to polychaete parapodia (Haeckel 1889). Although the similarity represents convergence, it is based on an underlying metameric architecture of serially repeated mesodermal segments produced by subterminal budding.

**Fig. 3:**
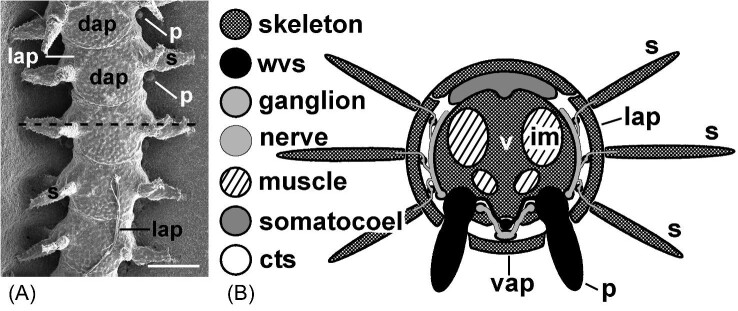
Serial repetition along the arm of a brittlestar. (**A**) External expression of serial repetition is seen in the arm of *Ophiactis savignyi*. Repeated parts include arms spines (s), the plates of each segment (dorsal arm plates [dap], lateral arm plates [lap], ventral arm plates [not in view]), and podia (p). Dashed line indicates the plane of the cross-section in frame (**B**). Scale bar 200 µm. (Scanning electron micrograph by Y. Demirci.) (**B**) Schematic of repeated elements of one segment. Triplet sets of arm spines (s) articulate on lateral arm plates (lap) of the skeleton. The bulk of each segment consists of a centrally located skeletal vertebra (v). Adjacent vertebrae are joined and operated by paired ligaments (not shown) and by dorsal and ventral intervertebral muscles (im). The radial canal of the water-vascular system (wvs) runs beneath the vertebra and sends a lateral water vessel to each podium (p), which protrudes externally between a lateral arm plate and the ventral arm plate (vap). The nervous system, within the connective tissue space (cts), includes segmental ganglia below the radial canal and gives rise to nerve tracts that connect to the podial ring ganglia and spine ganglia. For simplicity, the ectoneural and hyponeural nervous systems are not distinguished, and the hemal system is not shown.

Body parts of extant echinoderms are serially repeated along 5 axes, which confer the unique secondary symmetry on the group. The most obvious parts are spines, the podia (tube feet) of the water-vascular system, and the underlying major plates of an axis (Fig. 3A). Less obvious, except by dissection or histological sectioning, are the segmental nerves, muscles, ligaments, and elements of the hemal system (Fig. 3B). Although the pentaxial arrangement is obscured in some groups by branching (crinoids; Ausich et al. 1999, basketstars; Turner and O'Neill 2023) or by insertion of additional axes during postmetamorphic growth (multi-armed seastars; Hotchkiss 2000), the serial arrangement of parts along each axis is almost always apparent. Each segment remains distinct. There are 2 interesting variations. In the non-cidaroid sea urchins, the clustering or fusion of adjacent ambulacral plates creates a secondary serial repetition along the axes—a linear array of modules. The body wall of most sea cucumbers has lost the spines and underlying plates, and the rows of podia often are so crowded (or absent, as in the Apoda) that the segmental arrangement is lost. (Indeed, there seems to be no relationship between the ambulacra of sea cucumbers and those of other echinoderms [Mooi and David 1997; David and Mooi 1998; Udagawa et al. 2022].) The condition of the body in sea cucumbers explains their late placement within the phylum from among the worms (Hyman 1955). Fossil echinoderm groups that lack a pentaxial organization also demonstrate serial repetition of parts along their 1, 2, or 3 axes (Mooi and David 1998; David et al. 2000; Lefebvre 2003; Smith 2005).

The internal calcitic skeleton of adult echinoderms, the muscles and ligaments that bind and operate the component skeletal pieces (ossicles), the water-vascular system, and the hyponeural (motor) nervous system are derived from mesoderm (Hyman 1955; Giese et al. 1991; Heinzeller and Welsch 2001; Ruppert et al. 2004; Nielsen 2012). Each component contributes to a distinct segment within the ambulacral axis (Fig. 3B). This arrangement is modified in crinoids and holothuroids and within some subordinate taxa of other classes, but the derivation from mesoderm is uniform, except for the hyponeural nervous system of ectodermal origin in holothuroids (Mashanov et al. 2006). The ectoneural nervous system of ectodermal origin assumes the same metameric pattern. Interestingly, the gonads rarely are serially repeated except in crinoids (Holland 1991), some asteroids (Chia and Walker 1991), and an ophiuroid (*Ophiocanops fugiens*; Fell 1963).

**Fig. 4:**
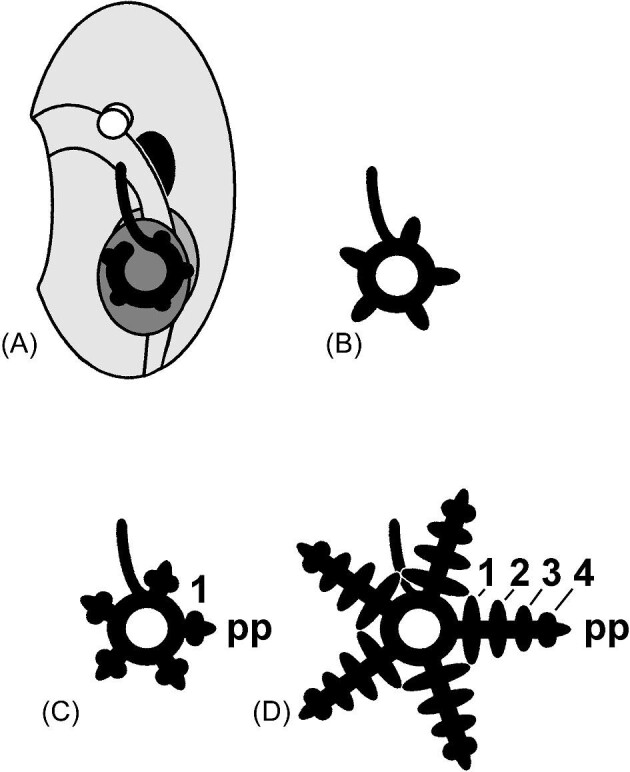
Early development of the water-vascular system in the juvenile rudiment (cf. Fig. 2A). (**A**) The left mesocoel encircles the early gut. (**B**) Five radial canals grow from the ring canal. (**C**) The growing tip of each radial canal is a primary podium (pp), in front of which is budded a succession of paired segmental secondary podia, the first pair (no. 1) shown here. (**D**) As the axis grows, additional podial pairs are added between the primary podium and the youngest pair (here, no. 4), the oldest pair (no. 1) lying nearest the mouth.

Subterminal addition of segments (metameres) appears early in growth of the juvenile rudiment (Fig. 4). The left mesocoel encircles a region around the future anterior region of the juvenile gut (Fig. 4A) and produces 5 primordia of the radial canals and primary (terminal) podium (Fig. 4B). As each radial canal elongates, pairs of secondary podia arise behind the primary podium, and the newest always in penultimate position (Fig. 4C–D). The 5 ambulacral axes grow meridionally and enclose the oligomeric embryonic or larval body as in echinoids (Fig. 2C), or they grow radially and extend from the central body (disk) as “arms” as in ophiuroids (Fig. 3A).

**Fig. 5:**
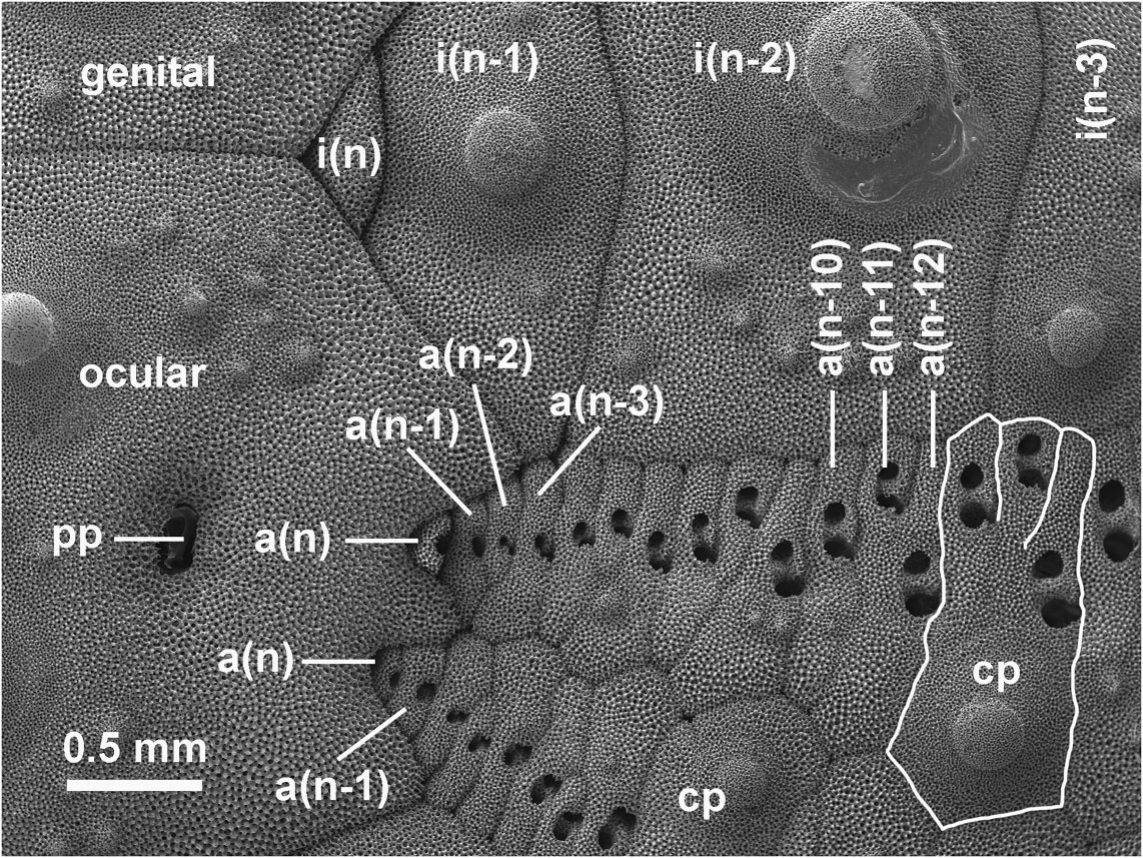
Subterminal addition of ossicles and podia in the sea urchin *Tripneustes ventricosus*. Near the top of the globe-like test, an ocular plate, bearing a single pore for the primary podium (pp), produces 2 columns of ambulacral plates (e.g., *a*[*n*], *a*[*n*-1], *a*[*n*-2] in one column) and shares production of the flanking interambulacral columns of plates (e.g., *i*[*n*], *i*[*n*-1], *i*[*n*-2]) with adjoining genital plates. As more ambulacral plates are added, they group together as triads in this species as compound plates (cp). Members of a triad eventually fuse together, as in the compound plate composed of a(*n*-10–*n*-12) and the next older compound plate outlined here for emphasis. The youngest (*n*th) externally visible ambulacral plate of one column has a simple podial pore and lies between the ocular plate and the next older ambulacral plate (*a*[*n*-1]). With growth of an ambulacral plate, the podial pore appears doubled on the surface. Ambulacral plates and compound plates of 2 ambulacral columns are staggered, forming a zigzag suture between the columns. Interambulacral plates in this species are larger than ambulacral and compound plates and are not in one-to-one correspondence; in this case, interambulacral plate *i*(*n*-2) shares sutures with ambulacral plates *a*(*n*-4–*n*-13). Soft tissue such as podia has been removed by treatment with bleach. Scanning electron micrograph.

This process of subterminal addition—long demonstrated to occur in echinoderms (Ludwig 1881; Jackson 1912; Hyman 1955; Thompson et al. 2021; Czarkwiani et al. 2022; Mashanov et al. 2022)—was incorporated into the ocular plate rule (OPR) of Mooi and David (1993), who later advanced the extraxial-axial theory (EAT; Mooi et al. 1994; Mooi and David 1998). The EAT distinguishes between those parts of the skeleton that are axial (derived from behind the ocular or terminal plate in the juvenile rudiment) and extraxial (derived more diffusely from the oligomeric embryonic or larval body). According to the OPR, elements of the ambulacral axis are formed in a budding zone between an unpaired ocular or terminal plate and the next younger ossicle (Fig. 5). The ocular “plate” (not always plate-like) and its unpaired primary podium are comparable to the pygidium in other metameric body plans. Appearance of segments begins early in the juvenile rudiment and continues throughout life. The elements (hemi-segments or antimeres) to the right and left of the bilateral axis are added oppositely (aligned in pairs, as in extant asteroids and ophiuroids) or alternately (in a staggered row, as in echinoids, crinoids, and some early fossil asteroids and ophiuroids) (Spencer and Wright 1966; Glass and Blake 2004) (Fig. 5). The aligned arrangement confers bilateral (reflection) symmetry on the axis, and the staggered arrangement confers glide-reflection symmetry (Kuznetsov 2024). The bilaterality in extant asteroids and ophiuroids is a derived state from the staggered arrangement of hemi-segments associated with left-right asymmetry from dexiothetism (left-side dominance due to right-side positioning [or left-side positioning; Holland 1988]) of ancestral echinoderms and chordates (Jefferies et al. 1996; Minguillón and Garcia-Fernàndez 2002). The distinction between production of hemi-segments in staggered and aligned rows is associated with expression of the *engrailed* regulatory gene along the ambulacral axis, resulting in the organization of the radial nerve into ganglia in extant asteroids and ophiuroids (Fig. 3B) and giving a staggered non-ganglionated arrangement in other groups (Wray and Lowe 2000). Although Mooi et al. (2005) argued that the plesiomorphic staggered pattern of echinoderm ambulacra makes the similarity of their metameric organization and expression of *engrailed* “superficial and because of convergence” compared with other metameric metazoans, the evidence for glide-reflection sinistrality in many deuterostome groups by Kuznetsov (2024) at least allows for homology among the deuterostomes.

Echinoderms are metameric animals: Serially arranged parts of mesodermal origin are produced in segments behind a terminal element (pygidium) along axes that form early in growth. Metamery obscures the oligomeric plan of the embryonic and larval body; thus, Hyman (1955) wrote that echinoderms are “without any body divisions corresponding to the [tripartite] coelomic divisions,” in contrast, for example, to acorn worms, which retain the prosoma (proboscis), mesosoma (collar), and metasoma (trunk) as adults (Fig. 2B–C). Whereas annelids, arthropods, most chordates, and some groups of fossil echinoderm (Lefebvre 2003; Smith 2005) are metameric with a monaxial body plan, extant echinoderms are metameric with a pentaxial body plan, possibly derived from replication of a metameric anteroposterior axis as in the hypotheses of Raff and Popodi (1996) and others (Morris 2012; Morris and Byrne 2014; Byrne et al. 2016, 2021; Isaeva and Rozhnov 2022); recent work of Formery et al. (2023) does not, however, support the replication hypothesis. (The view of rays as appendages or paramorphic homologs rather than as main axes still needs consideration [Hotchkiss 1998; Morris 1999; Minelli 2003b; Allievi et al. 2022; Isaeva and Rozhnov 2022], although it is not strongly supported by others [Heinzeller and Welsch 2001; Byrne et al. 2016; Formery et al. 2023].) The minimal contribution of extraxial parts of the body of echinoids (Mooi et al. 1994) gives them a clear oral-anal (oral-aboral) axis, the 5 nearly contiguous ocular plates defining the anal (or aboral) end as well as the distal end of the proximodistal axis (Minsuk et al. 2009) or anteroposterior axis (Morris 2009, 2012). In asteroids and ophiuroids, the great separation of the terminal plates (homologues of echinoid ocular plates) by multiplication of extraxial elements makes it difficult to recognize the posterior placement of the terminal plates on the proximodistal axis. Thus, echinoderms (e.g., asteroids) with the radial growth pattern of Fell (1962) appear as those (e.g., echinoids) of the meridional growth pattern in which the 5 axes have been separated and flattened out by the continued extraxial contribution of the oligomeric embryonic or larval body to postmetamorphic growth of the metameric adult. The holothuroids stand in contrast to these 2 forms in having lost metamerism in their primary axes, their primary podia remaining as the oral tentacles and not producing 5 series of subterminal, paired, and secondary podia (Udagawa et al. 2022); their bodies are, therefore, almost entirely extraxial (David and Mooi 1998; Mooi and David 2008) and exemplify severe reductions in the body plans as do echiurans and sipunculans among the annelids (Struck et al. 2007).

The echinoderm body results from 2 developmental modules: the embryo/larva and the juvenile rudiment (David and Mooi 1996; Minsuk and Raff 2002). An oligomeric “visceral” organization of embryonic/larval origin lies embedded within or superimposed upon a postmetamorphic, “somatic” body wall of 1–5 (or more) bilaterally symmetrical metameric axes (Fig. 2C), the “proximodistal axis” of Minsuk et al. (2009). This organization is comparable to, though not homologous to, Romer's (1972) view of the vertebrate body as a duality of external somatic and internal visceral “beings.”

## A history of neglect

It is the focus on the oligomeric plan of embryos and larvae that has obscured the metameric plan of adult echinoderms over the last century (or longer) despite the frequent description of subterminal addition of podia and ossicles to the proximodistal ambulacral axis in echinoderm literature. In the past few decades, studies in molecular genetics have both confirmed and altered our understanding of relationships among metazoan phyla and lower taxa, relationships originally based on body plans. The discovery more recently of the genetic basis underlying some morphology—for example, patterning along the anteroposterior and dorsoventral axes and left-right asymmetry—has led to a resurgence of interest in comparative studies of metazoan body plans (e.g., Raff 1996; Valentine et al. 1996; Arthur 1997; Conway Morris 1998; Budd 1999; Erwin 1999; Erwin and Davidson 2002; Minelli 2003a; Isaeva and Rozhnov 2021). It is fundamental to the synergy between molecular geneticists and morphologists to have a correct understanding of animal body plans; or, as in the case of echinoderms, we will fail to make comparisons, we will miss opportunities in collaboration, and progress in comparative animal biology will be impeded. A similar oversight of echinoderms in metazoan comparative anatomy was expressed by Formery et al. (2020) regarding evolution of the deuterostome nervous system because, as the authors argue, of the unusual pentaxial body plan.

Where would we be now in understanding those puzzling echinoderms had Hyman (1955) not concluded, “Neither do these coelomic subdivisions bring about any segmentation (by which is meant serial repetition) of any other structures. The echinoderms are not segmented animals.”? What different course might Clark's (1964) analysis of metamery and metazoan evolution have taken had he not dismissed echinoderms as unsegmented nor stated that “little of phyletic significance can be concluded from their structure.”? In a later paper Clark (1979) wrote, “If the vertebrates did not evolve directly from metameric invertebrates [then] the search for the earliest deuterostomes turns to the Echinodermata and Hemichordata.” Willmer (1990) pointed out that the discovery of the homeobox sequence in echinoderms derailed the association of the homeobox with metamery in the mid-1980s, as indicated in McGinnis's (1985) statement that “even a broadest definition of metamerism excludes the sea urchin body plan.. ..” Might Erwin and Davidson (2002) have proposed a common ancestor to protostomes and deuterostomes that was not quite so “very simply constructed” had they viewed echinoderms as metameric? A more recent treatment that all but excluded echinoderms from consideration was Minelli's (2003a) challenging discussion of metamery (segmentation) from an embryological viewpoint. The possibility of a monaxial metameric echinoderm was not considered by Chen et al. (2019) in speculating on the relationships of their new Ediacaran worm *Yilingia spiciformis*, which they offered as the earliest case of metazoan segmentation. And might echinoderms not be dismissed in their relationship to *Herpetogaster* of the Burgess Shale because of the segmentation of the latter (Nanglu et al. 2023)? Instead, Nanglu et al. (2023) suggested that *Herpetogaster* “may find some homology in the segmented bodies of early chordates.” The oversight of echinoderms as metameric probably derives from the emphasis on oligomery explained above, the pentaxial adult symmetry, and the lack of septation of the water-vascular system. Most recently, Formery et al. (2023) dismissed 4 hypotheses about evolution of the pentaxial body plan in their study of anteroposterior patterning genes; and they offered a new hypothesis, the “ambulacral-anterior model,” that viewed echinoderms as “mostly head-like animals.” Would that they had also addressed metameric arrangement of the axes, although Lacalli (2023) in reviewing their paper suggested that the evolution of segmentation post-dated a common deuterostome ancestor.

There has been occasional direct or indirect recognition of axial metamerism in echinoderms in the past 158 years. Haeckel (1866) advanced his idea (later named the Pentastraea Hypothesis; Haeckel 1889) that the monophyletic echinoderms evolved from annelids as a corm of metameric arms that were budded asexually within the larva and joined together at a common mouth. Haeckel (1889) proposed, however, that the 5 worms were joined at their tails, not their heads. Haeckel's view of the echinoderm body plan explains his inclusion of the phylum in his classic Tafel XII (Haeckel 1874) as a co-lineage with arthropods descending from “Ringelwürmer (Annelida).” Gislén (1930) described “secondary metamery” in fossil and extant echinoderms. These earlier, more extensive treatments have been followed by brief mention of metamery in echinoderms. In the caption of one figure, Pentreath and Cobb (1972) pointed to the arrangement of nerves in a “bilaterally and metamerically repeated series” along the echinoderm ambulacrum. Cobb (1995) later referred again to the repeated segmentation of the echinoderm radial nerve. Holland and Holland (1998) recognized the homalozoans to be segmented (but the extant echinoderms to be reduced and to “retain only embryonic trimery”). Morris (1999) postulated the origin of echinoderms from a bilateral metameric ancestor by reduction to a 3-segmented intermediate form, with subsequent radial outgrowth of appendages rather than axes. Although she described metameric growth of the appendages, she did not call them “metameric”; but she declared podia of the ancestral echinoderm to be “homologues of somites of metameric bilateral forms.” Heinzeller and Welsch (1999, 2001) described the segmental organization of echinoderm arms and considered their possible homology with the trunks of other metameric animals; but their analysis of homology was specifically focused on the hydrocoel/notochord and ectoneural tube/neural plate of echinoderms and chordates, complementing the proposal of Welsch (1999). Other authors have used the terms “metameric” or “segmented” and their variants in describing echinoderms (e.g., LeClair 1996; Czarkwiani et al. 2013, 2022; Formery et al. 2020; Thompson et al. 2021; Isaeva and Rozhnov 2022; Mashanov et al. 2022), although Ezhova et al. (2021) and Ezhova and Malakhov (2021, 2022) misapplied the term to coelomic stacking (Mooi et al. 2005).

In contrast to the occasional reference to metamerism or segmentation in the echinoderm body plan over the decades by various authors, there is the significant body of work by the late Valerie B. Morris and her colleagues, often published with titles that refer to the “enigmatic echinoderm body plan.” Based on their studies in molecular genetics and developmental biology of echinozoans and asterozoans, they have drawn many conclusions about the echinoderm body plan: that the metameric paired secondary podia are homologues of somites of other metameric animals (Morris 1999, 2009), and their morphogenesis is controlled by Hox genes *HpHox11/13*; that the mouth is anterior (Morris et al. 2004) and that the series of secondary podia define an anterior–posterior axis (Morris 2009, 2012); that the bilateral plane of the metameric ambulacral axis is homologous with the bilateral axis of other deuterostomes (Morris 2007; Morris and Byrne 2014); that 4 ambulacral axes arose from duplication of an ancestral ambulacral axis (Morris 2012; Morris and Byrne 2014; giving rise to the term “conjoined integrated ‘quintuplets’” of Mashanov et al. 2022); that non-contiguous metameric axes of asteroids are the ancestral configuration and that the contiguous arrangement in echinoids is derived (Byrne et al. 2016); that, whereas metamery is patterned by Hox genes, pentamery is patterned by *Nodal* and *BMP2/4* duplication (Byrne et al. 2016, 2021) and the Pax-Six-Eya-Dach gene regulatory network (Byrne et al. 2018); that metameric ambulacra are axes, not arms or appendages (Byrne et al. 2016), homologous with chordate somites (Morris 1999, 2012), contrary to Kuznetsov (2024); and that their morphological and genetic evidence contradicts the view of a dorso-ventral inversion of chordates among deuterostomes (Morris et al. 2009; Morris 2012, 2016), in agreement with Kuznetsov (2024).

## Concluding remarks

Most oligomeric phyla consist of sessile or sedentary organisms (Clark 1964; Nielsen 2012), and echinoderms are believed to have evolved from sedentary ancestors (Hyman 1955). Oligomery “suits” echinoderms well, and it is the body plan expressed earliest in echinoderm development. However, early ontogenesis is appended with metamery of later differentiation and growth. Unusually, metamery in echinoderms is expressed by the mesosoma, not the metasoma; the mesocoel does not become septate; and metamery is bi-, tri-, and pentaxial as well as monaxial among the several extinct and extant subphyla. Echinoderms certainly add to the diversity of metameric body plans in the Metazoa.

The EAT (David et al. 2000; Mooi and David 2000; Hotchkiss 2012) has helped to discern morphological homologies and phylogenetic affinities within the echinoderms. The work of Morris and her colleagues and of other workers mentioned above are changing our view of homologies within the deuterostomes. Perhaps now rethinking echinoderms as metameric animals by the broader community of students of the Metazoa will help us understand better the relationship of echinoderms to other deuterostome phyla and advance our knowledge of comparative biology, from regulatory genes to morphology.

In one (David and Mooi 1996) of a series of papers by Bruno David and Rich Mooi on their EAT, the authors concluded with the encouragement, “Such changes in the way we think about echinoderm morphology could deeply affect our exploration. . . of hypotheses for the origin of the phylum itself.” Welsch (1999) posed, “Of course, one has to ask, whether there are actually really new insights in respect of the echinoderm body plan.” He then provided evidence for “a possible homology between neural cord/notochord on the one hand and ectoneural cord/radial water vascular canal on the other.” Heinzeller and Welsch (2001) followed Welsch (1999) with the vision that “the entry [of echinoderms] into the club of true notoneuralians (i.e., deuterostomes with a dorsal neural tube) would be facilitated if the traditional view of the echinoderm body axis. . . is given up in favor of the concept of trunklike modules that hitherto were looked at as arms and, furthermore, if the ectoneural cord that dominates the nervous system of all classes except crinoids becomes substantiated as homologous to the chordate neural plate.” This first quarter of the 21st century does seem to be a time of new insights for the echinoderm body plan as Ernst Haeckel's then-new ideas provided in the last third of the 19th century. Comparative biologists and phylogeneticists of the Metazoa should re-evaluate the position of metamerism and other characters as ancestral features in deuterostome phylogeny (e.g., Nanglu et al. 2023, their Fig. 5). In the present “exciting time for the *Hox* field” and progress “to decipher the mysteries of *Hox* activity” with its complex of transcription factors, target genes, cofactors, and cooption to other functions in specifying segmental morphologies (Hueber and Lohmann 2008), we should not be hasty in making conclusions about homologies in features of body plans among the metameric echinoderm and other deuterostome groups, even with the most recent intriguing view of echinoderms as “head-like animals” (Formery et al. 2023).
